# Liver regeneration microenvironment of hepatocellular carcinoma for prevention and therapy

**DOI:** 10.18632/oncotarget.12101

**Published:** 2016-09-17

**Authors:** Hanmin Li, Lisheng Zhang

**Affiliations:** ^1^ Hepatic Disease Institute, Hubei Provincial Hospital of Traditional Chinese Medicine, Wuhan, People's Republic of China; ^2^ College of Veterinary Medicine, Huazhong Agricultural University, Wuhan, People's Republic of China

**Keywords:** liver regeneration, hepatocellular carcinoma, immunity, inflammation, vasculature

## Abstract

Research on liver cancer prevention and treatment has mainly focused on the liver cancer cells themselves. Currently, liver cancers are no longer viewed as only collections of genetically altered cells but as aberrant organs with a plastic stroma, matrix, and vasculature. Improving the microenvironment of the liver to promote liver regeneration and repair by affecting immune function, inflammation and vasculature can regulate the dynamic imbalance between normal liver regeneration and repair and abnormal liver regeneration, thus improving the microenvironment of liver regeneration for the prevention and treatment of liver cancer. This review addresses the basic theory of the liver regeneration microenvironment, including the latest findings on immunity, inflammation and vasculature. Attention is given to the potential design of molecular targets in the microenvironment of hepatocellular carcinoma (HCC). In an effort to improve the liver regeneration microenvironment of HCC, researchers have extensively utilized the enhancement of immunity, anti-inflammation and the vasculature niche, which are discussed in detail in this review. In addition, the authors summarize the latest pro-fibrotic transition characteristics of the vascular niche and review potential cell therapies for liver disease.

## INTRODUCTION

Liver cancer is one of the most common cancers worldwide and is known to affect several million individuals. Hepatocellular carcinoma is the most common type of liver cancer, accounting for 70%-85% of all liver cancer cases and representing the fifth most common malignancy and the third leading cause of cancer-related death worldwide. Approximately one million new cases are diagnosed every year with an almost equal number of deaths [1, 2, 3, 4]. As we know, chronic hepatitis is associated with unresolved inflammation, which contributes to liver injury and concurrent regeneration, giving rise to fibrosis, cirrhosis, and eventually HCC [4, 5, 6].

Research on liver cancer prevention and treatment has mainly focused on the liver cancer cells themselves. Currently, liver cancers are no longer viewed as only collections of genetically altered cells but as aberrant organs with a plastic stroma, matrix, and vasculature. The regeneration microenvironment is important for liver cancer prevention and therapy [7]. Improving the liver microenvironment to boost the regeneration of damaged liver cells by affecting immune function, inflammation and the vasculature may coordinate the dynamic imbalance between normal liver regeneration and repair and abnormal liver regeneration, thus improving the microenvironment of liver regeneration to prevent and cure liver cancer [6, 8, 9].

Surgical resection has been an effective method for the early treatment of liver cancer [10]. The liver has the greatest regenerative capacity of any organ in the body; thus, the microenvironment of the tumor or the resected liver is critical for patient survival. The 5-year liver transformation rate and the tumor recurrence rate are as high as 38% to 65%, and the 5-year survival rate is only 50% after complete surgical resection of the tumor and the metastatic region (RO resection) [11, 12, 13]. Some scholars have found that surgical resection promotes liver cancer recurrence and metastasis, suggesting that surgery may impact the liver regeneration microenvironment, causing the residual liver (micrometastases and de novo cancer) to disseminate and resurge [14, 15, 16]. In this review, we summarize the differences and similarities between the liver regeneration microenvironment and immunity, inflammation and the vascular environment during liver cancer development.

## IMMUNE MICROENVIRONMENT, LIVER REGENERATION AND LIVER CANCER

The immune microenvironment is an important component for liver tumor initiation and development, where a number of factors combine in a unique way to form the tumor immune regulation system. Hepatic Kupffer cells (KCs), natural killer (NK) cells, and NKT cells are innate immune effectors, and they cooperate to fight bacterial and viral infections as wells as liver cancers [17]. At the same time, body immunity directly affects liver regeneration. The innate immune system consists of an intricate network of interacting cells and cytokines and exerts significant effects on liver regeneration. Strey reported that the complementary C3a and C5a proteins, two potent inflammatory mediators of the innate immune response, participate in the priming phase of liver regeneration since the C3a and C5a knockout mice demonstrate impaired liver regeneration. [18, 19].

The most abundant hepatic lymphoid cell population consists of NK cells and NKT cells [20]. The cells exert effects on liver regeneration in animal models. After a 70% partial hepatectomy, the number of NKT cells increases in the remnant liver, and they are involved in the process of liver regeneration [21, 22]. Large amounts of cytokines produced by NKT cells have multiple functions in liver regeneration and immune responses [23]. NKT cell activation has been demonstrated in hepatitis B virus transgenic mice, and the depletion of both NKT and NK cells enhances liver regeneration post-PH, whereas the depletion of NK cells alone has no effect, revealing the negative regulation of activated NKT cells in liver regeneration [24, 25]. NKT cells are strongly activated by the lipid antigen α-galactosylceramide, resulting in a dramatic induction of Th1- and Th2-type cytokine expression (IFN-γ and IL-4, respectively) [26]. Yin illustrated that IL-4 KO mice exhibit restored liver regeneration by IL-4 mediated NKT cell expansion, thus increasing total IFN-γ expression [25]. All of the results suggest that NKT cells affect liver regeneration by influencing the inflammatory hepatic microenvironment.

NK and NKT cells are not only involved in innate immunity reactions against viruses and microbes but also act as potential agents in cancer immunotherapy [27]. Clinical observational studies have focused mostly on NKT cells, which are decreased in solid tumors; increased NKT cell numbers are associated with a better prognosis [28, 29, 30]. The proportion of NK cells is decreased in patients with hepatitis C virus (HCV) infection, and NK receptor expression and biological functions are upregulated, including cytokine production [31]. Several studies in animal models have demonstrated that the transfer of NK cells resulted in anti-tumor effects [32, 33, 34]. Invariant NKT (iNKT, type I NKT) protects mice from chemical induced liver injury and fibrosis [35, 36]. Usually, type I NKT cells play anti-tumor roles by producing interferon-γ (IFN-γ), promoting NK cell activation and killing tumor cells or tumor-associated macrophages, whereas type II NKT cells negatively regulate tumor immune surveillance [37].

Kupffer cells make up the largest population of macrophages in the liver. The analysis of structure and enzymology characteristics indicated that the Kupffer cell affects phagocytosis, secretion, immune regulation and surveillance [38]. Communication between Kupffer cells and monocytes is necessary for progenitor cell-mediated liver regeneration/repair. CD11b^+^ Kupffer/Mφ cells have been demonstrated to be involved in liver regeneration since the rate of division in these cells is dramatically increased via TNF/FasL signaling on the 3rd day of liver regeneration after PH [39]. However, the proportion of CD68^+^/Kupffer cells, the other macrophage cell type, is comparable after PH [40]. Abrogation of TNF-α using a neutralizing antibody or specific genetic down-regulation severely impairs liver regeneration/repair [41], suggesting that TNF is a very necessary component of liver regeneration.

Although the role of Kupffer cells in liver regeneration is still controversial, tumor-associated macrophages and T cells in the hepatic microenvironment are critical for liver regeneration in HCC. By using paracrine signaling, macrophages affect the proliferation of hepatic progenitors via TWEAK and influence progenitor cell fate through the Wnt signaling pathway. Extrahepatic macrophages are also recruited to the niche during liver regeneration in human diseases and animal models. Thus, the manipulation of liver regeneration with macrophages that affect progenitor cell expansion will be a very interesting and meaningful focus for future liver disease treatment [42, 43].

Dendritic cells (DCs) process and present antigens to T lymphocytes and activate naïve T cells to trigger the adaptive-immunity response against viral infection and tumor development. Under physiologic conditions, hepatic dendritic cells are immature and tolerant. However, in the chronically inflamed livers of hepatitis patients, dendritic cells mature and become proinflammatory. The expansion of DCs accelerates the regression of hepatic fibrosis. Furthermore, Henning et al. found that DCs (CD11c^+^ MHCII^+^ cells) restrict CD8^+^ T-cell expansion and limit toll-related protein expression and cytokine generation in a nonalcoholic steatohepatitis animal model [43, 44].

It is well known that hepatic stellate cells (HSCs) play critical roles in the process of liver fibrosis. Many studies have also found that HSCs have other multifunctional roles in protecting the liver, such as affecting liver regeneration, immune regulation and immune suppression. HSCs directly interact with NK cells, NKT cells and T cells. The activation of HSCs by microbiology products is mediated by TLR2, TLR4 and TLR9 to trigger proinflammatory responses [45, 46]. In summary, the activation of HSCs is critical for liver fibrosis and other hepatic diseases related to immune responses.

Taken together, anti-HCC immunity to improve the hepatic microenvironment may represent a promising treatment method.

## INFLAMMATORY MICROENVIRONMENT, LIVER REGENERATION AND LIVER CANCER

Chronic liver inflammation, often caused by hepatitis B virus (HBV) or hepatitis C virus (HCV) infection, plays a critical role in liver cancer initiation and development [47]. In most cases, the expression levels of cytokines such as interleukin-6 (IL-6) and TNF-α will increase [48, 49]. The priming phase of liver regeneration is stimulated by higher expression levels of TNF-α and IL-6, predominantly produced by non-parenchymal cells, along with activated STAT3 and NF-kB signaling pathways [50, 51]. Pikarsky reported that the prevention of NF-kB signaling in hepatocytes and early tumors lessened tumor aggregation in a mouse model [52, 53]. NF-κB activation is regulated by TNF-α produced by stromal cells [54]. Increasing the amount of TNF-α in the tumor niche induces hepatocytes to progress to malignancy [55, 56].

The NF-κB-mediated microRNA signaling pathway plays critical roles in human hepatic cell transformation and tumorigenesis in patient samples and animal models [57]. Additionally, chronic inflammation causes entirely spontaneous liver tumors in bile acid receptor–farnesoid X receptor (FXR) knockout mice at 15 months of age without toxin treatment, but not in FXR-gene-functional mice [58, 59]. Studies have also revealed that activated FXR inhibits NF-κB translocation and neutralizes the expression of an array of growth factors and cytokines [60].

**Figure 1:**
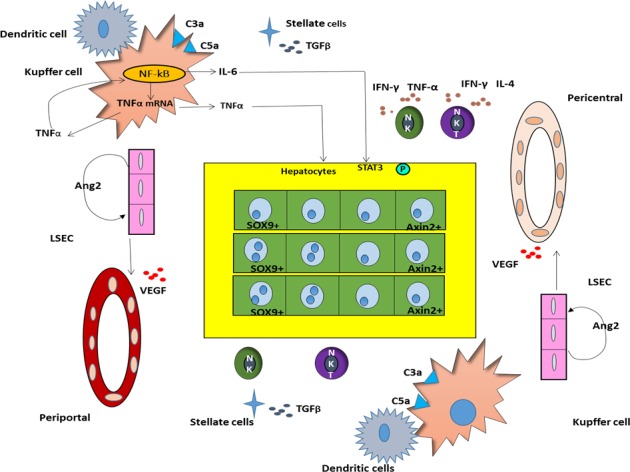
Microenvironment of immunosuppression, tumor-promoting inflammation, vascular reconstruction during liver regeneration of HCC

In addition to TNF-α, IL-6 is the other well-studied cytokine. IL-6 expression is elevated in liver tumors; IL-6 expression is non-detectable under physiological conditions [61, 62]. IL-6 exerts multifunctional roles via STAT3, which has been shown to be critical for the progression of HCC [63, 64, 65]. Other cytokines and growth factors, including IL-4, IL-10, IL-12, IL-13, IL-1a, IL-1b and IL-15, also play critical roles in the development of HCC [66, 67, 68, 69]. The cytokines not only enhance HCC tissue proliferation but also prime liver regeneration functions. The critical difference between liver regeneration-priming cytokine expression and cytokine expression in the HCC environment is that PH priming is under strict control, whereas the priming of HCC tissue and diseased liver tissue is not under strict control [70, 71].

Transforming growth factor beta (TGF-β) is a hepatocyte growth inhibitor. The immunosuppressive and proangiogenic effects of TGF-β1 in high concentrations are advantageous in the formation of the tissue microenvironment for liver cancer occurrence, in which the disruption of the TGF-β/Smads signaling pathway leads to disorders of the cell growth cycle and the occurrence of liver cancer [72, 73]. TGF-β1 fails to inhibit the proliferation of residual liver cancer cells when TGF-β receptor expression in hepatoma cells decreases, thereby inducing liver cancer recurrence and progression [74]. In HBV-triggered HCC development, the viruses and the malignant hepatocytes initiate the production of cytokines and growth factors such as IL-6, TNF and TGF-β. Meanwhile, the cytokines and growth factors activate immune cells, inducing the production of TNF and IL-6 by Kupffer cells. These cells become more activated, causing more inflammation and compensatory expansion, as well as the generation of reactive oxygen species to determine the hepatocyte mutation fate [75, 76, 77].

In summary, the inhibition of cytokine production, the control of macrophage infiltration rate, and the regulation of cells that produce cytokines and growth factors that transform normal hepatic cells, are important for HCC prevention and therapy.

## VASCULATURE MICROENVIRONMENT, LIVER REGENERATION AND LIVER CANCER

HCC is a hypervascular tumor mass. The malignancy regulatory node of the endothelial niche in HCC results from the imbalance of pro- and anti-angiogenic factors, and HCC angiogenesis causes the formation of dysfunctional vessels to promote liver disease progression. Combining tyrosine kinase receptor inhibitors that target endothelial cells and vascular smooth muscle cells/pericytes successfully diminishes tumor angiogenesis and decreases tumor size.

The liver is zonated. In the pericentral zone, Axin2^+^ pericentral cells are localized to a Wnt-rich anatomical niche, whereas Wnt-regulated genes, such as β-catenin and Apc, are known to contribute to liver development and zonation [78, 79, 80]. In the periportal zone, SOX9^+^ periportal hepatocytes are capable of extensive expansion and can restore liver mass after severe and chronic liver injuries, but these cells do not give rise to HCC [81]. Due to the exo-oncogenic expression in induced pluripotent stem cells (iPS) [82, 83] and the difficulty in obtaining fetal liver progenitor cells [84, 85], near periportal hepatocyte (SOX9^+^) cells have great potential for future clinical use to treat liver disease when proper isolation techniques and massive expansion protocols are established.

Hepatic sinusoidal endothelial cells (HSECs) line sinusoids to form an interface between the circulating blood and hepatocytes, and they are actively engaged in the clearance of various substances from the gut due to their high endocytic activity [86].

As the first sensor of dynamic changes in hepatic microcirculation and as the protective barrier against various substances and antigens passing through the liver microcirculation, HSECs play an important role in viral infection, liver damage, liver fibrosis, and tumor development. Moreover, HSECs have recently been demonstrated to be essential components in liver development and regeneration [87, 88]. Sinusoidal endothelial cells coordinate liver regeneration and angiogenesis, and spatiotemporal control of the intrinsic properties and paracrine activity of endothelial cells is critical in the modulation of different phases of regeneration [89].

Timing is crucial for growth factors to enhance regeneration and prevent maladaptive healing. The production of VEGF by hepatocytes peaks at 48-72 h after PH and is identified primarily in periportal hepatocytes [90]. When binding to the vascular endothelial growth factor receptor-1 (VEGFR-1) of hepatocytes, VEGF causes hepatocyte proliferation. VEGF induces the autocrine proliferation of hepatocytes. Hepatocyte proliferation also results from the paracrine regulation of hepatocyte growth factor (HGF) and IL-6 by HSECs [90, 91]. The activation of VEGFR-2 induces HSEC proliferation. The angiopoietin/Tie family, including angiopoietin 1 (Ang-1) and angiopoietin 2 (Ang-2), also plays a role in HSEC proliferation. The inhibition of Ang2 reduces vascular endothelial growth factor receptor-2 expression and attenuates the angiogenic functions of endothelial cells *in vitro* [92], suggesting that Ang2 is required for the angiogenic phase of liver regeneration.

The regulation of HCC angiogenesis is very complex and includes a variety of pro-angiogenic factors, anti- angiogenesis, and many other factors yet to be identified. The lack of lymphatic drainage in the center of the cancer, the difficulty of metabolite discharge, and the lowering of the pH also play roles in angiogenesis. The increased expression of VEGF has been shown to be positively correlated with arterial and sinusoidal capillary formation during HCC progression [93, 94, 95]. Microvessel density in liver cirrhosis samples is significantly lower than in liver cancer specimens, but samples of liver cirrhosis associated with hepatitis B or C infection contain a number of inflammatory mediators that could induce the expression of angiogenic cytokines, such as IL-8, TNF-α, TGF, bFGF, VEGF. These factors may also indirectly induce other angiogenic factors to stimulate the formation of microvasculature.

Signaling from the vascular niche plays an important role in both liver regeneration and malignancy, but the factors determining the balance in cell signaling remain unknown. Ding's results showed that chemokine receptors are crucial for the balance in mouse models [88]. Ding reported that pro-regenerative CXCR7-encoding transcripts increased in HSECs in response to acute injury. The CXCR4 signaling pathway was activated, and CXCR7 signaling was suppressed in chronic liver injury models. During chronic liver injury, a lack of CXCR7 and an increase in CXCR4-dependent transcript signaling in HSECs causes the progression to fibrosis [88]. Thus, timing may be crucial when selectively enhancing regeneration and preventing maladaptive healing.

The HCC vasculature can be classified into two major types of vessels based on morphology: capillary-like microvessels and sinusoid-like vessels. HCC patients with sinusoid-like vessels have a shorter survival time, although the microvessel density within a tumor with a sinusoid-like vasculature is significantly lower than the density of a tumor with capillary-like microvessels [96, 97].

Since angiogenesis is critical for the maintenance of tumor growth, progression and metastasis, anti-angiogenic drugs have shown promise as anti-HCC therapeutics and may become the focus of anti-tumor research. Several anti-liver cancer drugs that target angiogenesis, such as bevacizumab, sorafenib, and gefitinib, have achieved some results. However, angiogenesis is a complex physiological and pathological process, and these drugs cannot completely block the construction of tumor microvessels.

Indeed, vessel co-option is a recently accepted concept for the development of tumor vasculature in solid tumors, and HCC is one of the tumors heavily dependent on co-opted vessels [97]. Moreover, vessel co-option is one of the reasons that liver cancer is resistant to sorafenib treatment. Vessel co-option, a process of hijacking the blood vessels in the surrounding normal tissue along with the invasion of a solid tumor, has been perceived as an important means to create a tumor vasculature [98, 99].

Altogether, the combination of targeting blood vessels with conventional anticancer drugs, cytotoxic drugs, and a more in-depth understanding of the molecular mechanisms of angiogenesis will provide new avenues for the comprehensive understanding and treatment of HCC in the future.

In conclusion, the deterioration of the liver regeneration microenvironment causes changes in immunity, inflammation and the vascular microenvironment, thereby promoting HCC occurrence and development. The improvement of the microenvironment during liver regeneration with the regulation of multi-component, multi-target, multi-level, multi-channel and multi-timed factors may provide new strategies for liver cancer prevention and treatment.
